# Microchambers with Solid-State Phosphorescent Sensor for Measuring Single Mitochondrial Respiration

**DOI:** 10.3390/s16071065

**Published:** 2016-07-09

**Authors:** Ted D. Pham, Douglas C. Wallace, Peter J. Burke

**Affiliations:** 1 Department of Biomedical Engineering, University of California, Irvine, CA 92697, USA; tedp@uci.edu; 2 Center for Mitochondrial and Epigenomic Medicine, University of Pennsylvania, Philadelphia, PA 19104, USA; wallaced1@email.chop.edu; 3 Department of Electrical Engineering, University of California, Irvine, CA 92697, USA

**Keywords:** respiration, mitochondria, oxygen consumption rate, phosphorescent probes, PtTFPP

## Abstract

It is now well established that, even within a single cell, multiple copies of the mitochondrial genome may be present (genetic heteroplasmy). It would be interesting to develop techniques to determine if and to what extent this genetic variation results in functional variation from one mitochondrion to the next (functional heteroplasmy). Measuring mitochondrial respiration can reveal the organelles’ functional capacity for Adenosine triphosphate (ATP) production and determine mitochondrial damage that may arise from genetic or age related defects. However, available technologies require significant quantities of mitochondria. Here, we develop a technology to assay the respiration of a single mitochondrion. Our “micro-respirometer” consists of micron sized chambers etched out of borofloat glass substrates and coated with an oxygen sensitive phosphorescent dye Pt(II) meso-tetra(pentafluorophenyl)porphine (PtTFPP) mixed with polystyrene. The chambers are sealed with a polydimethylsiloxane layer coated with oxygen impermeable Viton rubber to prevent diffusion of oxygen from the environment. As the mitochondria consume oxygen in the chamber, the phosphorescence signal increases, allowing direct determination of the respiration rate. Experiments with coupled vs. uncoupled mitochondria showed a substantial difference in respiration, confirming the validity of the microchambers as single mitochondrial respirometers. This demonstration could enable future high-throughput assays of mitochondrial respiration and benefit the study of mitochondrial functional heterogeneity, and its role in health and disease.

## 1. Introduction

Mitochondria play a multitude of roles in cell life and death [1,2,3]. In cell life, mitochondria produce the majority of cellular energy Adenosine triphosphate (ATP) and sequester harmful reactive oxygen species; in cell death, mitochondria play an active role in the so-called “intrinsic” pathway of apoptosis or programmed cell death [3,4]. It logically follows that mitochondrial dysfunctions resulting in defective energy metabolism and resistance to apoptosis have been linked to various disorders such as Alzheimer’s disease, cardiovascular diseases, diabetes, Parkinson’s disease, and cancer [5]. To assess mitochondrial dysfunction, current state-of-the-art experiments often report the mitochondrial respiration rate because it directly reflects the functional status and the health of the mitochondrion [6].

Convincing evidence exists that mitochondria have genetic heteroplasmy at multiple levels: as an organism ages, genetic (especially mitochondrial DNA based) defects in mitochondrial function become more prevalent, resulting in a heterogeneous distribution of mitochondrial DNA within the organism and within a given tissue [7]. During germline maturation, defective mitochondria are screened through unknown mechanisms to prevent offsprings from carrying serious mutations [8]. While technologies for assaying genetic variation among mitochondria are relatively mature [9,10], technologies to measure *functional* heteroplasmy are still lacking. One of the most significant functional assays, the quantification of the oxygen consumption rate, is usually reported as an average of thousands of cells or isolated mitochondria [6,11]; hence, the dynamics and variability of respiration rate from single cells or single mitochondria are lost. Furthermore, rare cell behavior such as stem cells or circulating tumor cells [7,12,13] is masked in such an ensemble approach.

To this end, we have developed a technology to assay the respiration of a single mitochondrion. Our “micro-respirometer” consists of micron sized chambers etched out of borofloat glass substrates and coated with an oxygen sensitive phosphorescent dye Pt(II)-meso-tetra(pentafluorophenyl)porphine (PtTFPP) mixed with polystyrene. The chambers are sealed with a polydimethylsiloxane (PDMS) layer coated with oxygen impermeable Viton rubber to prevent diffusion of oxygen from the environment. As the mitochondria consume oxygen in the chamber, the phosphorescence signal increases, allowing direct determination of the respiration rate. Experiments with coupled vs. uncoupled mitochondria showed a significant difference in respiration, confirming the validity of the microchambers as single mitochondrial respirometers. This demonstration could enable future high-throughput assays of mitochondrial respiration and benefit the study of mitochondrial functional heterogeneity, and its role in health and disease.

## 2. Materials and Methods

### 2.1. Mitochondrial Isolation

The mammalian cell line HeLa (American Type Cell Culture) was maintained in the log growth phase using minimum essential medium (MEM) supplemented with 10% fetal bovine serum (FBS). All cell culture related supplies were obtained from Thermofisher Scientific (Waltham, MA, USA). 10^7^ cells were typically harvested for mitochondria isolation.

Before isolation, the confluent cells were stained with 100 nM MitoTracker Green FM for one hour. The cells were then washed with fresh media.

Our complete isolation buffer contains 225 mM mannitol, 75 mM sucrose, 0.5 mM EGTA, 20 mM HEPES, 0.5% (*w*/*v*) Bovine Serum Albumin (BSA), 1X protease inhibitor, pH 7.2 with 1 M KOH. All buffer chemicals were purchased from Sigma Aldrich (Saint Louis, MO, USA). The stock isolation buffer was prepared without BSA and protease inhibitor and stored at 4 °C. Mitochondria from the cultured cells were isolated using differential centrifugation. After collection, the cells were transferred to a glass homogenization tube in 3 mL complete isolation buffer and homogenized with 30 strokes on ice. The cells were then transferred into 2-mL Eppendorf tubes and centrifuged at a low speed of 2000 × *g* for 4 min at 4 °C. The resulting supernatant was collected and centrifuged at a high speed of 12,000 × *g* × 10 min at 4 °C. The supernatant as well as the light-colored fluffy sediment containing damaged mitochondria were aspirated and the resulting pellet was resuspended in respiration buffer (140 mM KCl, 2 mM MgCl_2_, 10 mM NaCl, 0.5 mM EGTA, 0.5 mM KH_2_PO_4_, 2 mM HEPES, 5 mM succinate, 2 μM rotenone, pH 7.2 adjusted with KOH). However, for mitochondria protein analysis, the mitochondria were resuspended in KCl buffer without EGTA. The protein analysis was done with BCA assay kit supplied by Thermo Fisher Scientific, and the result indicated a typical yield of 140 µg mitochondrial protein. As a quality control, we used a Hansatech Oxytherm to measure the respiratory control ratio of a typical mitochondrial preparation with this protocol and found the ratio to be around 3.1, which is good for cultured cells [14].

### 2.2. Microchambers Fabrication

**Figure A3:**
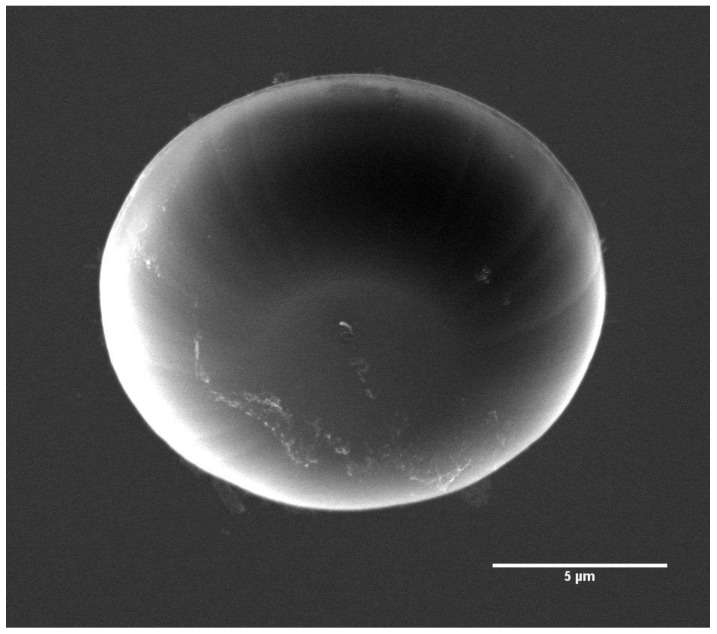
A scanning electron microscope (SEM) image of a microchamber.

Borofloat glass wafers (3″ × 3″ × 0.7 mm) with a pre-deposited 120 nm Cr layer were supplied by TELIC Company (Valencia, CA, USA). On top of the Cr layer, there was also a 530 nm preprocessed layer of AZ1500 positive photoresist. Using photolithography, Cr etching, and 48% HF wet etching, we created microchambers in the borofloat glass substrate with a depth of 7.5 µm and a surface diameter of 15 µm. The chamber’s depth was measured by a Dektak 3 surface profilometer. A scanning electron micrograph of one microchamber is shown in Figure A3.

The oxygen-sensitive dye was obtained from Frontier Scientific (Logan, UT, USA) (Catalog # PtT975) and was incorporated with polystyrene (PS), average M_w_ 280,000 Sigma Aldrich (Saint Louis, MO, USA) (Catalog# 182427). The PtTFPP/PS oxygen sensing mixture was prepared in toluene with 150 µM PtTFPP and 64 µM PS final concentrations. A few drops of this mixture were quickly transferred onto the substrate containing the microchambers using a cotton swab and the wafer was heated at 120 °C for 1 min. Immediately after the wafer was sufficiently cooled, we used scotch tape to extract the dye from the wafer surface, leaving PtTFPP only at the bottoms of the microchambers. Due to the capillary effect [15], the deposited dye formed a ring around the bottom of each chamber.

### 2.3. PDMS and Viton-Coated Top Insulating Layer

PDMS was prepared according to the manufacturer’s (Dow Corning, Midland, MI, USA) recommendation in 10:1 ratio with the curing agent. The PDMS were prepared into 3 cm × 2 cm × 3 mm slabs. Viton rubber was obtained from Pelseal Technology (Bensalem, PA, USA) (PLV 2000) company and mixed with its accompanying accelerator #4 in 44:1 ratio. Viton was paint brushed onto the top and the sides of each PDMS slab and allowed to cure at room temperature (25 °C) overnight.

### 2.4. Oxygen Calibration and Sealing Test

An inverted microscope Olympus IX71 was used to capture the phosphorescent intensity from PtTFPP/PS when exposed to 0% O_2_ (from 100% N_2_ standard laboratory line), 21% O_2_ (composition of O_2_ was assumed from the standard laboratory house air line), and 100% O_2_ (from an industrial cylinder). The air was flown approximately 5 cm from the top of the devices. Each gas was connected to a separate tubing and could be switched quickly. The microscope was equipped with a 395 nm excitation LED and the appropriate filter cube for 650 nm emission capture by QImaging QIClick camera.

To test the effectiveness of the insulating layer, oxygen sealing test was carried out by periodically exposing the microchambers with 100% O_2_ and measuring the changes in the phosphorescent intensity. Three tests were performed: (1) Microchambers; (2) Microchambers covered with a PDMS slab; (3) Microchambers covered with a Viton coated PDMS slab. Measurements were performed in the dark at room temperature (25 °C).

### 2.5. Experimental Procedure

Experiments were done at the UCI optical biology core with a Zeiss LSM 780 confocal scanning microscope. Excitation wavelengths were 405 nm and 488 nm by lasers for PtTFFP/PS and Mitotracker Green, respectively. The LSM 780 can simultaneously monitor both red and green channels. Exposure time was set at 200 ms and images were taken every one second.

Microscope focus to the bottom of the chambers were first achieved before mitochondria deposition. Following the addition of 50 µM ADP to the mitochondrial suspension to initiate respiration, 2 µL of 0.2 µg/mL of mitochondrial protein was dropped and spread to the wafer’s surface. After the mitochondria were deposited, a Viton coated PDMS slab was quickly placed on the wafer, effectively sealing and separating the microchambers. We then performed image acquisition for the durations specified in the results. To decouple the mitochondria or to induce maximal respiration rate, carbonyl cyanide 3-chlorophenylhydrazon (CCCP) was added along with ADP. Measurements were performed in the dark at room temperature (25 °C).

Up to 15 chambers were visible in the field of view. The chambers were separated into two groups: (1) with mitochondria and (2) without. Image processing was done with ImageJ. The average red intensity and green intensity (when applicable) were measured for each chamber. The data were processed in IGOR software. To minimize the effect of photobleaching, the red intensity was divided by the average green intensity from all applicable chambers. These data were further normalized by the initial processed intensity. The linear fit feature in IGOR was used to fit a line to the plotted data.

## 3. Results

### 3.1. Device Characterization

**Figure 1:**
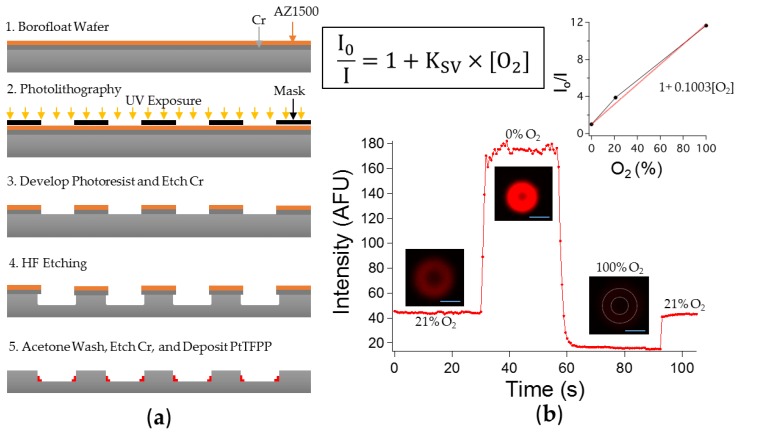
(**a**) Overview of the device fabrication scheme; (**b**) Testing the oxygen sensitivity of a device by measuring the phosphorescent intensity. Three gas lines were used: 21% O_2_ (house air), 0% O_2_ (100% N_2_), and 100% O_2_. The top left inset is the Stern-Vollmer equation, outlining the inverse relationship between the intensity and the oxygen concentration with I_0_ representing the intensity at 0% O_2_. Images of the microchamber were set at the same contrast level. At 100% oxygen, the red intensity was indiscernible at the chosen contrast level so an outline of the chamber was included. Top right inset is the plotted average measured intensities (I_0_/I with I_0_ being the intensity at 0% O_2_) at three different oxygen concentrations and the fitted line for Stern-Vollmer relationship. Scale bar is 10 µm.

We fabricated our microchambers using a simple photolithography and wet etching process. To minimize oxygen permeability, we picked borofloat, which is generally considered oxygen impermeable. The process is briefly overviewed in Figure 1a. First, we replicated the surface (top) geometry of the microchambers from a predesigned photomask to the photoresist. After a few etching steps, the top glass surface of the microchambers was exposed and a depth could be created with 48% HF etching. One minute 48% HF etch created on average a 7.5 µm depth in the borofloat wafer. The etch undercut expanded the diameter of our chambers’ top surface from 10 µm (mask design) to 15 µm. Finally, the PtTFPP dye incorporated in polystyrene were deposited at the bottom of the microchambers. To test the behavior of our sensing PtTFPP/PS layer, we exposed the layer to light streams of gas containing different concentrations of oxygen. Similarly, we also tested the effect of the Viton coated PDMS slab on sealing the top by quantifying the signal changes under alternating normal air and 100% oxygen gas. These tests were performed with an inverted microscope (Olympus IX71, Olympus America Inc., Center Valley, PA, USA) equipped with an LED excitation source at 395 nm and a long pass emission filter at 650 nm.

#### 3.1.1. Sensitivity to Oxygen: Oxygen Quenches PtTFPP Phosphorescence

We directly flew normal air (21% O_2_), 0% O_2_, and 100% O_2_ sequentially at about 5 cm above the top of the microchambers. The gas lines could be switched quickly because we used individual tubings. A base line was established first with normal air (21% O_2_). The response of our fabricated PtTFPP/PS sensing layer was as expected. The higher the oxygen concentration, the lower the phosphorescence intensity. Figure 1b shows the respective intensities under normal air, 0% oxygen, and 100% oxygen. The intensity and oxygen concentration could be fitted with a Stern-Vollmer linear relationship which shows that the intensity is inversely proportional to the oxygen concentration. We observed some delays (5–6 s) from 21% oxygen to 0% oxygen and to 100% oxygen and 1 s delay switching from 100% oxygen to 21% oxygen. Even though, these time delays could seem fast, they are expected because the time constant of PtTFPP is within the microseconds range [16,17], and when factoring experimental errors from manual gas switching, the time delays were reasonable. In summary, these observations show that our microchambers were indeed sensitive to oxygen.

#### 3.1.2. Effect of Viton Coating

**Figure 2:**
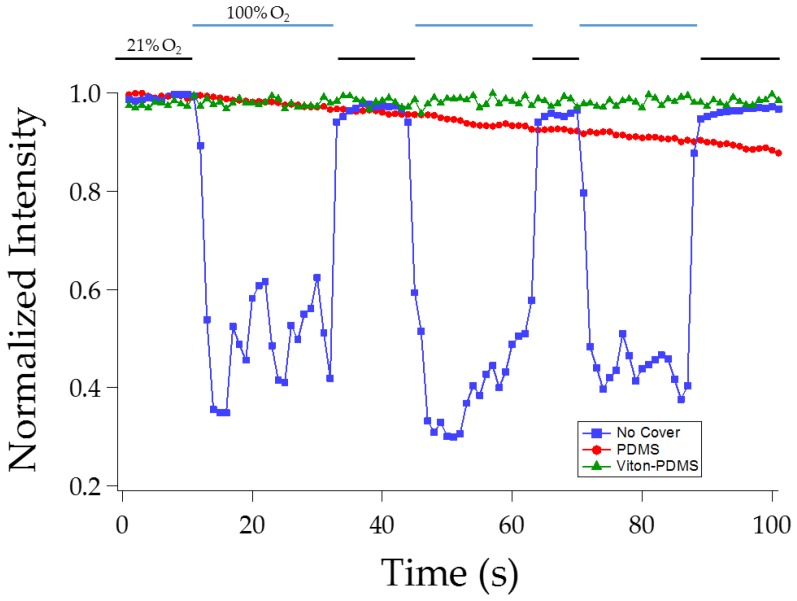
Normalized intensity (measured intensities divided by the corresponding maximum intensity) vs. time to summarize the effect of covering the chambers with 1. No Cover; 2. A PDMS slab; 3. A PDMS slab coated with Viton rubber. The microchamber was exposed to alternating normal air (21% O_2_) and 100% O_2_ gas. At each exposure outlined by the top two lines, multiple measurements were taken as indicated by the data points.

To seal the top of the chamber, we tested a flexible sealing layer made of Viton covered PDMS. Viton is a black fluoroelastomer with exclusive impermeability to gases including oxygen. Viton has been used previously in microfluidics but mainly for chemical resistance instead of gas impermeability [18]. Viton’s permeability to oxygen ranges from 0.6 to 1.3 Barrer (10^−10^ cm^3^·cm·s^−1^·cm^−2^·cmHg^−1^) [19,20], significantly lower than that of PDMS which is 781 Barrer [21]. The use of Viton, which is black, also prevents light leakage from excitation source and maximizes the acquisition of the emitted light. Figure 2 shows that under intermittent 21% and 100% oxygen exposure, minimal change was observed in the phosphorescence intensity from the chamber covered with Viton-coated PDMS. In contrast, the uncovered microchamber exhibited intensity fluctuations during the gas exchange test. Minimal delay, in fractions of a second, in gas exchange was achieved by using different tubings for the gas streams. Adding a layer of PDMS on top of the microchamber partially sealed the sensing layer from being directly exposed to 100% O_2_. However, because PDMS is still oxygen permeable, repeated exposure to 100% oxygen allows gradual migration of oxygen into the chamber resulting in the observed gradual decrease in signal intensity.

### 3.2. Single Mitochondrial Respiration

After demonstrating the oxygen sensitivity of our microchambers and the sealing capability of the Viton-PDMS layer, we performed respiration experiments on individual mitochondria. For these experiments, we utilized a confocal microscope LSM780 (Zeiss, Pleasanton, CA, USA) which can monitor both the red phosphorescence from PtTFPP and the green fluorescence from Mitotracker Green used to stain the mitochondria. To minimize the effect of photobleaching, we used the ratio between the green and the red intensity as the metric and further normalize the signal to the initial reading.

#### 3.2.1. Chambers with Mitochondrion vs. Empty Chambers

**Figure 3:**
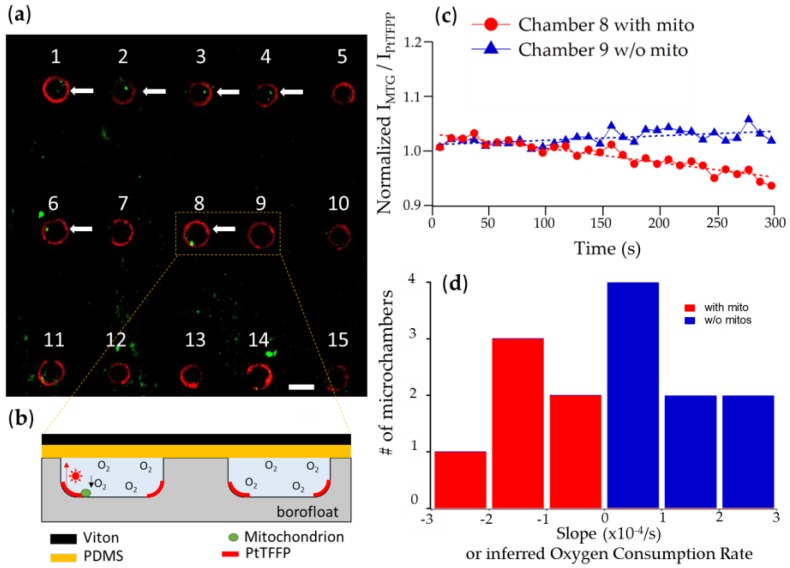
(**a**) Up to 15 microchambers could be viewed in the same field of view. After mitochondria deposition and Viton-PDMS sealing, some microchambers contained mitochondria (indicated by the white arrows) and others did not. Scale bar is 15 µm; (**b**) Zoomed in, side-view cartoon of chambers 8 and 9; (**c**) Normalized intensity (radiometric measurement divided by the initial measurement) measurement of a representative microchamber with mitochondria and another chamber without mitochondria. The result for chamber 8 indicates a gradual decrease in signal signifying a depletion of oxygen while the result for chamber 9 displays a small positive slope probably due to unaccounted photobleaching. Data points were averaged every 10 s; (**d**) The histogram showing the calculated slopes of the measured intensities from chambers 1 to 14. Chambers 1, 2, 3, 4, 6, 8 were the ones with mitochondria. Chamber 15 was not included in the analysis due to insufficient signal readout. The slopes are directly related to the oxygen consumption rates.

Figure 3 summarizes representative results of a single mitochondrial respiration experiment. This experiment was done in triplicate with two successful trials. Figure 3a shows that up to 15 microchambers could be viewed in the same field of view. Mitochondria were distributed randomly, thus some chambers contained mitochondria while others did not. Our results show that in the chambers with mitochondria, the signal (the inverse of PtTFPP intensity) gradually decreased. This decrease means that oxygen was being depleted from the chambers, so we reasoned that the oxygen was being consumed by our deposited mitochondrion and this resulted in a slight decrease in the signal readout. The microchambers without any mitochondria, on the other hand, exhibited a slight increase in the signal readout, which we attributed to residual photobleaching not fully accounted for by the ratiometric method. Figure 3c shows the representative signal readout from a microchamber with a mitochondrion and another without; data points were averaged every 10 s in Figure 3c curves. Consequently, the oxygen consumption rate by individual mitochondria can be roughly represented by the slope at which oxygen was being depleted in individual microchambers. Figure 3c summarizes the distribution of such slopes or deduced oxygen consumption rates, with the negative slopes representing oxygen consumption rate from microchambers with a mitochondrion and vice versa with the positive slopes indicating empty microchambers. From the six microchambers with a mitochondrion, the inferred rates of respiration fluctuated (Figure 3c), indicative of functional heterogeneity even at this modest level. The scalability of our microchambers then make our technology an attractive platform for high-throughput study of mitochondrial functional heterogeneity. One potential complication in our measurement is that because the microchambers all interact with the PDMS slab’s bottom surface, which is not coated with Viton, diffusion of oxygen from one chamber to the neighboring ones could be possible, but our results showed this effect could be ignored. There were also mitochondria outside of the chambers because the deposition was nondeterministic. These mitochondria were most likely pressed between the glass surface and the PDMS. Since PDMS is hydrophobic and we observed that the sealing layer adhered well to the glass surface, we are confident that most of the experimental liquid were inside the microchambers (Figure 3b). Similar observations were also reported in single cell respiration experiments [22].

#### 3.2.2. Chambers with One Mitochondrion vs. Two vs. One Treated with CCCP

**Figure 4:**
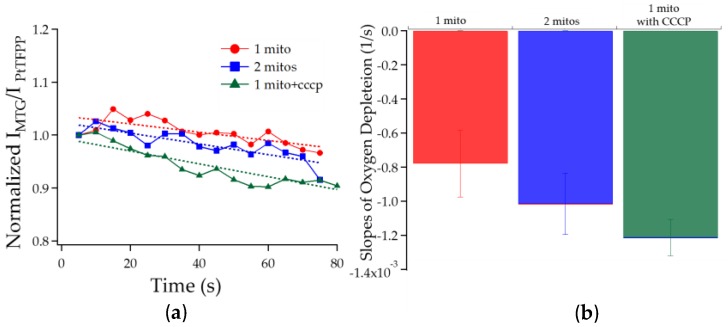
(**a**) Plotted PtTFPP normalized phosphorescent intensity from chambers with one mitochondrion, with two mitochondria, and with one mitochondrion treated with CCCP. Data points were averaged every 5 s; (**b**) The slopes of the fitted lines in (**a**) are plotted. Results indicated that the rates of oxygen depletion in the corresponding microchambers. The errors were obtained from fitting the data.

To further substantiate our demonstration of single mitochondrial respirometers, we compared the oxygen depletion in a chamber with one mitochondria against one chamber with two mitochondria and another with one mitochondrion treated with CCCP. As expected, the rate at which oxygen was being depleted in the chamber with two mitochondria was higher than that in the chamber with only one mitochondrion. Furthermore, CCCP is known to decouple the electron transport chain and ATP synthase, allowing the electron transport chain to run at its maximal rate. This translates into more oxygen being reduced at complex IV to water. We expected the oxygen depletion rate in chambers with CCCP treated mitochondrion would be higher than the non-treated counterpart and our result confirmed this expectation (Figure 4). This experiment was done on the same mitochondrial preparation and devices from the same batch. The calculated slopes here differ from those in Figure 3 because the devices used were from two different batches. Even though devices’ behaviors are similar within a batch, from one batch to another, to accurately compare data, we would need a more robust calibration method (Supplemental Information).

## 4. Discussion

We have demonstrated a simple, robust technology to assay respiration of individual mitochondria. The proof of concept shows that measurement of functional heteroplasmy (that is, functional differences between mitochondria) is possible. This is significant because heteroplasmy in the mitochondrial DNA content has been shown, but techniques to assay the functional differences are poorly developed.

How does this compare to existing technology? Broadly speaking, two methods are dominantly used to measure respiration: Electrochemical (Clark type electrodes) and luminescent readouts. Oxygen sensors based on a Clark-type oxygen electrode rely on the amperometric electrochemical reaction between a coated Pt-electrode and dissolved molecular oxygen, and have challenges for miniaturization such as consumption of oxygen by the sensor itself, and the requirement for a reference electrode [14,23,24,25,26,27]. Our approach in this paper has been to use oxygen-sensitive phosphorescent probes, which do not consume oxygen and offer high sensitivity, specificity, fast response times down to milliseconds [28], and demonstrated compatibility with biological samples [29].

Table 1 summarizes the features of selected state of the art commercially available instruments (Oroboros [24], Warner [25,30], Hansatech [31], Seahorse [6,11], MitoXpress [32,33]), as well as those demonstrated in research labs (Cantebury [34,35],MIT [26], and UWash [22,36]).

Due to the diffusion of O_2_, measuring the absolute oxygen consumption rate of any biological sample requires a completely sealed and isolated chamber. The available commercial Clark-type electrode systems offer excellent sealing chambers but require a large amount of sample and as discussed previously, they are not suitable for miniaturization. For the systems based on phosphorescent probes, commercial companies offer alternatives to sealing chambers. MitoXpress uses a layer of mineral oil to minimize to back diffusion of O_2_; however, this approach is limited to assessing treated versus non-treated sample and still does not solve the sealing problem. Seahorse Biosciences, on the other hand, employs a semi-closed chamber and develops a complex mathematical model to calculate the O_2_ back diffusion, which is deducted from their final measurements. Oxygen sensing has also been integrated in microfluidic channels [34,26,37]; yet, these channels were designed to monitor the oxygen level in tissue cultures and not for the purpose of detecting oxygen consumption rate. Single-cell respiration with a set of sealed micro-wells have been demonstrated by UWash [38], however, this setup requires an external pressure by a piston. Our microchambers, in contrast, require no piston for sealing and can achieve single mitochondrial resolution, which translates to approximately 1 pg worth of mitochondrial protein [39]. In addition, our micro-chambers are only 1.5 pL, the smallest in size to our knowledge with the next smallest being 80 pL from UWash. These attributes present considerable improvements of our microchambers over the compared technologies.

While our approach demonstrates proof of concept, additional work is required to make the technology broadly useful in studies of mitochondrial heteroplasmy. First, the noise of the system needs to be critically assessed to determine the ultimate limits of respiration. At this point, while the integration time required for measurement (~10 s) is slightly better than existing technologies (~one minute), the signal to noise is inferior. One method to improve this would be to use phosphorescence lifetime rather than intensity measurement, which typically suffers from lower drift and improved signal to noise. We have demonstrated the feasibility of this approach for measuring oxygen tension in our chambers (Supplemental Information), so this is a clear possible next step. Second, a method needs to be developed to more practically introduce reagents into the chamber over time, rather than seal the chamber only once for the entire assay. Third, the instrument needs to be built out for turnkey operation, rather than requiring a skilled operator. This should be straightforward with appropriate off the shelf electronics, optics, and control software. Finally, the technology needs to be validated against existing biological models. Such a validation demonstration, of course, would only be possible for ensemble respiration measurements, as biological models for functional heteroplasmy of mitochondria are only now possible to assess based on the prototype devices presented here. Of course, these four additional tasks are beyond the scope of this proof of concept prototype demonstration.

Intracellular probes for measuring oxygen concentrations have recently advanced in single cell applications [40,41,42]. However, these probes are still a matter for further research [17].

## 5. Conclusions

Overall, we have developed a proof-of-concept microchambers capable of detecting respiration at the single mitochondrial level. We achieved this by etching the microchambers out of borofloat glass (an oxygen impermeable material), depositing a phosphorescent sensing layer made of PtTFPP and polystyrene, and applying a sealing layer made of Viton coated PDMS to minimize oxygen diffusion.

## Figures and Tables

**Table 1:** Comparison to selected current respirometers.

Company/Group	O_2_ Reporter	Detection Method	Chamber Size	Sealed?	Fluid Handling	Adhesion?	# of cells	Amount of mitos	Throughput	Cost
Oroboros[24]	Clark	Electrical	2 mL	X	X	-	10^6^	1 mg	low	high
Warner [25,30]	Clark	Electrical	100 µL	X	-	-	-	20 µg	low	mid
Hansatech [31]	Clark	Electrical	1 mL	X	-	-	10^7^	5 mg	low	mid
Seahorse [6,11]	Luminescence	Intensity	5 µL	-	X	X	-	10 µg	high	high
MitoXpress [32,33]	Luminescence	Intensity, Lifetime	200 µL	X	-	-	-	60 µg	high	mid
Canterbury [34,35]	PtOEPK	Intensity	-	X	X	X	-	-	-	low
MIT [26]	PtOEPK	Lifetime	20 nL	-	X	-	-	-	-	low
Uwash [22,36]	Pt-porphyrin	Lifetime	80 pL	X	-	-	1	-	mid	low
This work	PtTFPP	Intensity	1.5 pL	X	-	-	-	1 pg	mid	low

**Figure A1:**
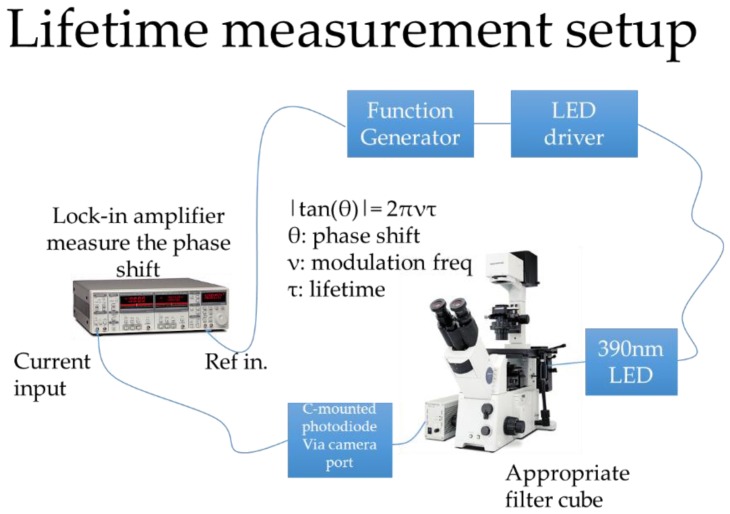
Photobleaching independent measurement setup for the phosphorescent lifetime of PtTFPP oxygen sensitive dye. The phase shift between the signal from the photodiode and the source excitation wavelength can be used to calculate the lifetime.

**Figure A2:**
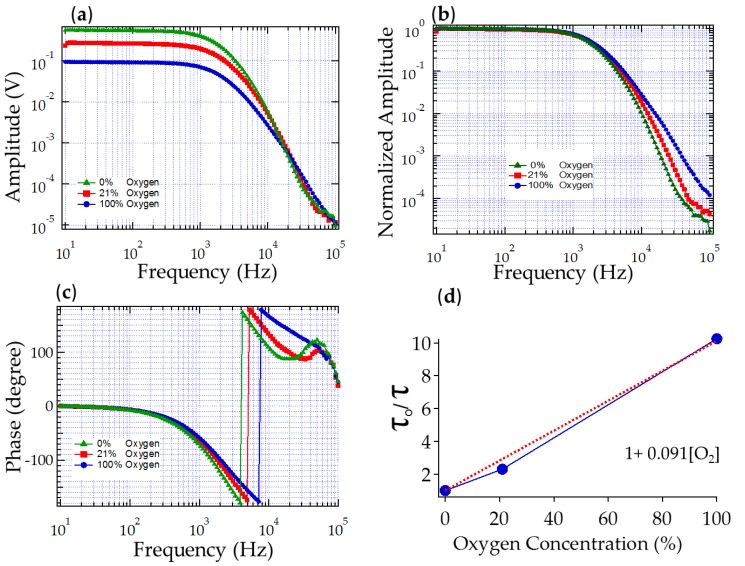
Spectral characterization of the oxygen sensing microchambers. (**a**) Amplitude was measured in V for three oxygen concentrations; (**b**) Normalized amplitude vs. frequency; (**c**) The phase vs. frequency behavior. The lock-in SR830 displays phase output from −180° to 180° hence the jump at around 3 kHz; (**d**) Due to a considerable difference in the phase shift, we chose 10 kHz as the reference frequency and calculate the phosphorescent lifetimes from the phase readouts. The plot of τ_o_/τ exhibits a linear Stern-Vollmer relationship. Our measured lifetime for 0%, 21%, 100% O_2_ are 43.7 µs, 19.0 µs, and 4.26 µs, respectively. All of these values are consistent with what have previously been reported [16,17].

## Abbreviations

The following abbreviations are used in this manuscript:

PDMS	polydimethylsiloxane
PtTFPP	Pt(II)-meso-tetra(pentafluorophenyl)porphine
PS	polystyrene
OCR	Oxygen consumption rate

## Apendix

### A.I. 1: Qualitative vs. Quantitative Measurement of Respiration

What this study has not accomplished is to quantify the absolute oxygen consumption rate. In order to do that, careful calibrations of oxygen concentrations in liquid must be carried out. The calibrations done with simple gas streams were only to demonstrate the sensitivity of the sensing layer. However, the detection of single mitochondrial respiration was done in solutions, reflecting the relative oxygen concentrations in liquid rather than in air. Hence, to obtain an accurate calibration of the sensors, we will need to expose the microchambers to solutions with known oxygen concentrations. Another way to improve on the existing detection method is to utilize the phosphorescent lifetime of PtTFPP instead of the intensity. The sensing principle of the oxygen-sensitive phosphorescent probes (PtTFPP included) is based on the dynamic quenching of the dyes’ phosphorescent intensity and lifetime [43]. Specifically, oxygen molecules collide with the luminophores, deactivating them from their lowest excited state to the ground state [44]. The relationship of this quenching effect is described by the Stern-Volmer equation:

(A1) $$\frac{I_{o}}{I}=\frac{\tau_{o}}{\tau }=1+K_{sv}\left[O_{2}\right]$$

$I_{o}$ and $\tau_{o}$ are the reference values in the absence of oxygen, whereas I and τ are the measured intensity and lifetime. The ratio of $\frac{I_{o}}{I}$ and $\frac{\tau_{o}}{\tau }$ are expressed linearly in terms of the Stern-Volmer constant $K_{sv}$ and the concentration of O_2_ in the solution. Equation (A1) is alternatively expressed in terms of the partial pressure of oxygen, which is related to the concentration of O_2_ in solution via Henry’s law. Typical oxygen assays based on phosphorescent dyes will begin with constructing a calibration curve for three to five different concentrations of oxygen with the choice of detected signal of either phosphorescent intensity or lifetime. If the selected measurement is intensity, the problems with photobeaching and optical interference from biological samples should be considered. As the probe’s phosphorescence degrades after repeated exposure with excitation light source, the intensity-correlated measurement of oxygen concentration becomes unreliable. To mitigate this effect, one can utilize a concurrent oxygen-insensitive dye [45] (in our case, Mitotracker green). Conversely, phosphorescent lifetime is immune from photobleaching and optical interference because the lifetime, which is the average time the luminophore stays in the excited state, is an inherent property of each probe. We have developed a preliminary setup to detect the phosphorescent lifetimes of PtTFPP and the results are promising. By relying on the phosphorescent lifetime, we could completely eliminate the effect of photobleaching and could also operate free from a microscope.

The system is outlined in Figure A1 and the preliminary measurement is summarized in Figure A2.

### A.I. 2. Proper Interpretation of Single Mitochondrial Respiration Data

Mitochondria vary in size, shape, mtDNA content, and thus (presumably) function [46,47]. Although extensive technology exists to measure their size, shape, and mtDNA content, technology to assess mitochondrial functional heterogeneity is still lacking. This has motivated our goal of assaying heterogeneity in mitochondrial function. In fact, ours is the first technology to determine functional respiration of intact, vital mitochondria. Therefore, this is a method to determine function, and may lead in the future to understanding of structure-function relationships of individual mitochondria. Unfortunately, such a comparison of single mitochondria is still not possible. In traditional ensemble experiments, the respiration rate is typically normalized to the total “amount” of mitochondria, typically measured as the amount of mitochondrial protein measured by a BCA assay. However, such a normalization is not possible in our case, since the technology to measure the amount of protein in a single mitochondrion does not exist yet. A careful study of volume of mitochondria might be possible in future generations of this technique using advanced light microscopy, but has not been developed in this work. Advanced electron microscopy (such as TEM microscopy [47]) can determine structure, but the mitochondria are not functional once they have been fixed. Nonetheless, our technique can measure variation from one mitochondria to the next, which no other technique can do. In contrast to existing technology which allows for study of mitochondrial *content* (*typically mtDNA*) or *structure* (typically with ultra-high resolution electron microscopy), this work opens a new window to understanding and studying variability in biological *function* from one mitochondria to the next.

For our purpose, we would assume that all mitochondria have similar size and shape. The same assumption was implied in measuring the functional heterogeneity of membrane potential in individual mitochondria [46,48].

### A.I. 3. Lighting Conditions

Because mitochondria are also sensitive to light exposure [49], all mitochondria in this study were exposed to the same amount of excitation light with a dark isolated measurement environment during the experiments with confocal microscopy, which is still a recognized standard in studying single mitochondrial dynamics [50].
